# Characterizing Ligands that have Immunomodulatory potential at the Choroid Plexus in Neurodegenerative Disease

**DOI:** 10.1002/alz.089569

**Published:** 2025-01-03

**Authors:** Bhavya Bakshi, Katelyn Rosenbalm, Michael Tiemeyer

**Affiliations:** ^1^ Medical College of Georgia, Augusta, GA USA; ^2^ University of Georgia, Athens, GA USA

## Abstract

**Background:**

Inflammatory cells play a key role in the pathophysiology of AD and other neurodegenerative disorders. Glycans are known to mediate inflammatory cell activation and migration yet very little is understood about the expression of glycans, glycoproteins, and other glycoconjugates at the CP which serves as a gateway for peripheral immune cells into the brain. In a familial AD mouse model, we observed increased expression of Siglec‐F‐recognized glycans on CP epithelial cells. Siglec‐F is part of the Siglecs family, that modulate the immune responses by binding sialylated glycans.

**Method:**

To explore Siglec interactions with CP cells, we explored glycomics and glycoproteomics in CP papilloma cells (HIBCPP), which mimic the CP environment. We collected proteins secreted from HIBCPP cells and performed western blot analysis using Siglec‐Fc recombinant proteins to identify counter‐receptors. Secreted glycoproteins carrying Siglec‐F and ‐9 (the human functional orthologue of mouse Siglec‐F) ligands were observed at molecular weights that were similar to ligands harvested from choroid plexus organoid lysates. To characterize the nature of the glycans on the secreted counter‐receptors, we used specific endo‐glycosidases to digest the glycoproteins and performed biorthogonal analysis.

**Result:**

Analysis revealed keratan sulfate ligands for Siglec‐9 and Siglec‐F. Proteomics identified Galectin‐3 binding protein (Gal‐3BP) as a major carrier whose function in CP and CNS biology is unknown. In cancer and viral infections, Gal‐3BP has been shown to modulate immune and inflammatory responses, indicating that it may serve similar immunomodulatory effects in the CNS and at the CP via specific glycan‐mediated interactions at cell surfaces.

**Conclusion:**

The identification of Gal‐3BP and the glycans it carries as Siglec counter‐receptors creates new opportunities to explore the potential inflammatory role of glycans in neurodegenerative diseases like AD.

